# Experimental Warming Decreases the Average Size and Nucleic Acid Content of Marine Bacterial Communities

**DOI:** 10.3389/fmicb.2016.00730

**Published:** 2016-05-23

**Authors:** Tamara M. Huete-Stauffer, Nestor Arandia-Gorostidi, Laura Alonso-Sáez, Xosé Anxelu G. Morán

**Affiliations:** ^1^Plankton Ecology and Pelagic Ecosystem Dynamics, Centro Oceanográfico de Gijón/Xixón, Instituto Español de OceanografíaGijón/Xixón, Spain; ^2^Marine Research Division, AZTISukarrieta, Spain; ^3^Division of Biological and Environmental Sciences and Engineering, Red Sea Research Center, King Abdullah University of Science and TechnologyThuwal, Saudi Arabia

**Keywords:** marine bacteria, coastal ocean, flow cytometry, size, nucleic acids, temperature-size rule

## Abstract

Organism size reduction with increasing temperature has been suggested as a universal response to global warming. Since genome size is usually correlated to cell size, reduction of genome size in unicells could be a parallel outcome of warming at ecological and evolutionary time scales. In this study, the short-term response of cell size and nucleic acid content of coastal marine prokaryotic communities to temperature was studied over a full annual cycle at a NE Atlantic temperate site. We used flow cytometry and experimental warming incubations, spanning a 6°C range, to analyze the hypothesized reduction with temperature in the size of the widespread flow cytometric bacterial groups of high and low nucleic acid content (HNA and LNA bacteria, respectively). Our results showed decreases in size in response to experimental warming, which were more marked in 0.8 μm pre-filtered treatment rather than in the whole community treatment, thus excluding the role of protistan grazers in our findings. Interestingly, a significant effect of temperature on reducing the average nucleic acid content (NAC) of prokaryotic cells in the communities was also observed. Cell size and nucleic acid decrease with temperature were correlated, showing a common mean decrease of 0.4% per °C. The usually larger HNA bacteria consistently showed a greater reduction in cell and NAC compared with their LNA counterparts, especially during the spring phytoplankton bloom period associated to maximum bacterial growth rates in response to nutrient availability. Our results show that the already smallest planktonic microbes, yet with key roles in global biogeochemical cycling, are likely undergoing important structural shrinkage in response to rising temperatures.

## Introduction

The reduction in organism size with increasing temperatures is currently being considered as a possible third universal response to warming (Gardner et al., 2011) after mounting evidences of changes in seasonality (Visser and Both, 2005) and northward displacement of species distributions (Perry et al., 2005; Benito Garzón et al., 2009). This phenomenon is commonly referred to as the *Temperature Size Rule* (TSR, Atkinson, 1994). Initially, the changes in size with temperature were observed coupled to latitude and described at the inter-specific level for endothermic metazoans (Bergmann, 1847) in what has been known as the Bergmann’s rule. Presently this phenomenon has been widely observed both inter- and intra-specifically (James rule after James, 1970), in endotherms and in ectotherms (from where the term TSR actually originates, Atkinson, 1994), from unicells to metazoans and plants (Forster et al., 2011, 2013; Gardner et al., 2011; Hessen et al., 2013). A simple corollary of the TSR is that organism size and ambient temperature are negatively correlated, with organisms being usually smaller at higher temperatures.

The reason for body shrinkage at higher temperatures (or enlargement at lower temperatures) seems to be a suite of metabolic advantages for the organisms at the temperature they are experiencing (Daufresne et al., 2009). However, it has been argued that temperature *per se* may not be the direct trigger of the size-temperature variability, with other factors such as food availability and quality, oxygen diffusion or size-selective predation playing more important roles (Daufresne et al., 2009; Gardner et al., 2011). Moreover, it is highly plausible that the major forcing factors differ at different levels of organism complexity (Forster et al., 2011; Ohlberger, 2013). Although we lack a general explanation for the mechanisms responsible for this body size change, the pattern described by the TSR seems to be universal, although not free from exceptions, as discussed for instance in Sheridan and Bickford (2011) and Hessen et al. (2013).

In unicells, the reduction of size with temperature has been documented in multiple studies, both in laboratory experiments (e.g., Atkinson et al., 2003; Forster et al., 2013) and from *in situ* observations (e.g., Daufresne et al., 2009; Hilligsøe et al., 2011; Morán et al., 2015) with suggested explanations including mechanisms operating at ontogenetic (Forster et al., 2011) and ecological (Gardner et al., 2011; Forster et al., 2013) levels. For example, the reduction of size with temperature may be consequence of increased growth rates in which time between generations is reduced and cells do not reach maximum volumes (Brown et al., 2004; White et al., 2007; Sheridan and Bickford, 2011). In the same line, phenotypic plasticity can also contribute to size decrease as a mechanism to increase the surface to volume ratio in warmer waters to increase gas exchange and nutrient uptake (Hessen et al., 2013). In turn, ecological processes can explain the reduction of size with temperature as a shift in community composition toward smaller organisms (Daufresne et al., 2009).

More recently, and at the evolutionary level, the TSR has been also associated to changes in genome size, particularly in prokaryotes (Sabath et al., 2013), since organism and genome sizes have been shown to covary (Gregory, 2001; Hessen et al., 2013). Yet, the cause and evolutionary advantages for this association remain unclear. A selection toward streamlined genomes not explained by genetic drift has been observed for free-living, thermophilic bacteria (Sabath et al., 2013). However, at less extreme temperatures, there are also examples of streamlining of genomes of free-living marine bacteria such as *Prochlorococcus* (Dufresne et al., 2005) and the SAR 11 (Giovannoni et al., 2005) or SAR86 (Dupont et al., 2012) clades, all three ubiquitous in the oceans but particularly dominant in subtropical and tropical waters, where the highest (up to 32°C, e.g., Red Sea) ocean surface temperatures are found. Thus, the long-term streamlining in these taxa may be the result of a combination of high temperature and low nutrients in rather stable environments (Lauro et al., 2009; Yooseph et al., 2010). The putative genetic loss and size reduction associated with higher temperatures is relevant for marine prokaryotes due to their importance in biogeochemical cycles (Azam and Malfatti, 2007; Fuhrman, 2009). Recently, the hypothesis of a shift toward smaller marine organisms with global warming (Daufresne et al., 2009) has gained support for heterotrophic prokaryotes (Morán et al., 2015).

In this paper we explored whether the reduction in the average cell size and nucleic acid content (NAC) of marine bacterial assemblages operates at the short-term scale (days) in response to experimental warming. We performed 12 experiments in 2012 over a complete annual cycle with incubations at ambient temperature and 3°C above and below that value. We used flow cytometry to study the temporal evolution of the temperature-associated reduction in both size and NAC of two bacterial groups characterized by low (LNA) and high (HNA) relative NAC (Gasol et al., 1999) due to their widespread presence in all aquatic ecosystems (Bouvier et al., 2007). Additionally, we used CARD-FISH (Pernthaler et al., 2002) of major phylogenetic bacterioplankton groups and ARISA (Fisher and Triplett, 1999) to examine changes in community composition and test for the possibility of species substitution within the incubations under different temperature treatments. We show that the temperature responses in cell size and NAC of bacterioplankton communities were more pronounced during meso- to eutrophic periods, apparently linked to the changes in oceanographic conditions that characterize temperate marine ecosystems.

## Materials and Methods

### Study Site and Experimental Setup

Samples were collected form a continental shelf station in the southern Bay of Biscay (NE Atlantic), located at 37 km off the coast of Gijón/Xixón (43.675°N, 5.578°E). Surface (1–3 m) water was collected every month during 2012 (except the April sampling, which was delayed due to bad weather until May 2nd) from R/V “José de Rioja” with 5 L Niskin bottles in a rossette sampler attached to a SeaBird25 CTD probe.

Collected seawater (80 L) was divided into two treatments: 40 L were pre-filtered with 200 μm filters, to remove mesozooplankton, and placed directly into 20 L acid-washed Nalgene bottles for the whole community (C) treatment. The same volume was pre-filtered through a 0.8 μm cartridge for the filtered (F) treatment, to isolate heterotrophic prokaryotes (mainly bacteria) from phytoplankton and protistan grazers. Samples taken on site and analyzed through flow cytometry, showed that filtration removed 98 ± 1% (mean ± SE) of heterotrophic flagellates (93% considering autotrophic flagellates) but also removed on average 14 ± 3% of bacteria. Upon arrival (within 4 h from collection), water was subdivided into triplicate 4 L acid-washed Nalgene bottles and placed into three incubators programmed at three different temperatures (collection temperature, 3°C above and 3°C below the collection value) with light-dark hours mimicking the photoperiod of the sampling day. Therefore, each experiment consisted of a total of 18 bottles, with three replicates for each combination of temperature and treatment.

We followed the evolution of heterotrophic bacteria inside our bottles by sampling once or twice a day until the bacterial abundance reached a steady state or begun to decline, which took place between 4 and 7 days after the beginning of the experiment.

### Flow Cytometry: Bacterial Populations, Side Scatter, and Green Fluorescence

We shall use the term marine bacteria since archaea at this sampling site only contributed on average ≤ 1% to total marine prokaryotes (Alonso-Sáez et al., 2015). Flow cytometry samples were collected and analyzed according to Gasol et al. (1999). Samples consisted of 180 μL of water fixed with a final concentration of 1% paraformaldehyde and 0.05% Glutaraldehyde, frozen in liquid nitrogen and stored at -80°C until analysis within 4 months of collection at most (usually <1 month). Samples were processed in a BD FACSCalibur flow cytometer equipped with an air cooled blue (488 nm) argon laser. The previously frozen samples were thawed and stained with Sybr-Green I (Molecular Probes) at a final concentration of 10x. After 10 min in the dark, samples were run for 2 min at low speed (15–20 μL min^-1^). This fluorochrome bonds specifically to nucleic acids, which can be measured as green fluorescence intensity (Marie et al., 1999). Simultaneously, we collected information on side scatter (SSC), a proxy for individual size that measures the 90° angle light scattered by each cell when impacted by the laser. 1 μm fluorescent beads (Molecular Probes) were added to each sample and used as an internal standard to obtain relative units (ru) of the intensity of the green fluorescence and side scatter signals.

On a plot of green fluorescence intensity against side scatter we separated two bacterial populations according to differences in their NACs, named HNA and LNA bacterial groups after High Nucleic Acid and Low Nucleic Acid, respectively. We used the green fluorescence signal relative to the beads values (fru) directly as a measure of NAC but side scatter was further transformed to volume: first converted to diameter following an empirical calibration for the study area (Figure 1 in Calvo-Díaz and Morán, 2006) and then transformed to volume assuming a spherical shape for all cells.

### Bacterial Cell Size and NAC Comparison of Experimental and Time-Series Data

Along with experimental data collected for this study we used data from a 10 years time-series collected in the same sampling area between April 2002 and March 2012. Given that these samples were analyzed using a different nucleic acid stain (Syto 13, Molecular Probes), we conducted a calibration between both fluorochromes (Syto 13 and Sybr-Green I) by running twice a set of 21 samples and comparing the green fluorescence values obtained with each dye. The calibration samples were collected as part of the routine time-series sampling and included the whole range of sizes and fluorescence values considered for this study. The results are shown in the Supplementary Figure S1. The data of the time-series were transformed to Sybr-Green I values following this calibration.

### Bacterial Size, NAC, and Growth Rate Response to Temperature

To analyze the response to temperature of size and NAC, we used the mean value for each replicate bottle over the course of the experiment. In parallel to changes in abundance due to growth, cell sizes changed within the incubation from collection time to the end, generally with small increases during the lag phase, decreases during the exponential growth phase, and stabilization at the final stages. We chose to use the mean value after testing that there were minor differences between the mean cell size during the experiment, the mean cell size during the growth phase and the cell size found at the maximum abundance. Furthermore, for the June–September period, the LNA bacterial group in the C treatment did not show positive growth, and using the mean cell size during the experiment was the only way to obtain a value that we could use for all the experiments. To be consistent, we used the mean experimental NAC as well. The selection of the mean experimental size or NAC is further justified because we were interested in differences between experimental temperatures, not in the specific experimental dynamics. Differences within treatments were always lower than among treatment, both for size and NAC.

The slopes of the OLS linear regressions between the mean cell size and NAC for each replicate vs. incubation temperature (*n* = 9) were finally used to describe the temperature responses of each variable. A negative slope indicated that bacterial cell size or NAC decreased with increasing temperature while a positive slope indicated the opposite (cell size or NAC increased with temperature increase). These slopes were used to estimate the percent reduction in cell size or NAC for 1°C temperature increase following Atkinson et al. (2003), by normalizing them to ambient values rather than to 15°C (differences between normalization to 15°C or initial value were non-significant, paired *t*-test *p* = 0.5 for size and *p* = 0.75 for NAC, *n* = 48). In addition, we converted NAC fluorescence relative units (fru) to base pairs units using the rough approximation that the bimodal distribution of bacterial genome sizes with peaks at 2 Mbp and at 5 Mbp (Koonin and Wolf, 2008) are equivalent to our LNA and HNA flow cytometric groups, respectively. This assumption seems not far from real marine genomes since a typical LNA representative such as the SAR11 strain HTCC1062 has a genome size of 1.54 Mbp (Giovannoni et al., 2005) while HNA representatives such as *Roseobacter* or *Flavobacteria* have genome sizes ranging from 4.2 to 4.7 Mbp (Pinhassi et al., 2006; Kalhoefer et al., 2011). We calculated the decrease in Mbp per °C in the same way as with size or NAC explained above, but equaling the initial mean ambient NAC values of LNA and HNA bacteria in all experiments in 2012 to the aforementioned values of 2 and 5 Mbp.

The response of growth rate to temperature was calculated as the activation energy (*E*) following Brown et al. (2004) detailed in Huete-Stauffer et al. (2015). Briefly, growth rates were calculated as the slope of the linear phase of the natural logarithm of abundance against time in days (calculated by OLS) and *E* was calculated for each month and bacterial group as the slope of ln-transformed growth rates at each experimental temperature against 1/kT (known as an Arrhenius plot). Positive slopes indicate that growth rates increased with increasing temperatures.

### Bacterial Community Structure

CAtalyzed Reporter Deposition Fluorescence *In Situ* Hybridization (CARD-FISH) was performed following the modified protocol of Pernthaler et al. (2002) to estimate the relative abundance of four major phylogenetic groups of marine bacteria (Giovannoni and Stingl, 2005; Alonso-Sáez et al., 2015): SAR11 (Alphaproteobacteria), *Rhodobacteraceae* (Alphaproteobacteria), Gammaproteobacteria, and Bacteroidetes. Separate aliquots of the same samples were collected for CARD-FISH and flow cytometry analyses. Samples for CARD-FISH were collected daily from two of the three replicate bottles per treatment, and analyzed at the point of maximum bacterial abundance in the incubations. Briefly, 4.5 mL samples were fixed at room temperature for 3 h with 3.7% formaldehyde solution, filtered with 0.2 μm polycarbonate filters (Millipore GTTP 25 mm), dried and frozen at -80°C until further processing. Thawed filters were dipped in agarose 0.1% (w/w), permeabilized in lysozyme 10 mg mL^-1^ (Sigma) at 37°C for 1 h and incubated in achromopeptidase solution at 37°C for 30 min. For hybridization, filter sections were submerged overnight at 35°C in horseradish peroxidase (HRP) labeled probes (50 ng μL^-1^) diluted in 900 μL of hybridization buffer prepared with 45% formamide concentration for the SAR11 probe (SAR11-441R, Morris et al., 2002) and 55% for the *Rhodobacteraceae* probe (Ros537, Eilers et al., 2001), the Gammaproteobacteria probe (Gamm42a, Amann et al., 1990) and the Bacteroidetes probe (CF319, Amann et al., 1990). Amplification was performed using tyramide labeled Alexa 488 dye (37°C, 30 min) and filters were transferred onto slides and stained with 4′,6-diamidino-2-phenylindole (DAPI) at 1 μg mL^-1^. DAPI and CARD-FISH stained bacteria were quantified using an epifluorescence microscope (Leica DM5500B), a monochromatic camera (Leica DFC360 FX) and the ACMETool2 automatized image analysis software (technobiology.ch, Zeder et al., 2010).

Relative abundances (percentages) of each phylogenetic CARD-FISH group were calculated from the total DAPI abundances. The contributions of each group were used to analyze changes in community structure at a broad phylogenetic level.

Automated Ribosomal Intergenic Spacer Analysis (ARISA; Fisher and Triplett, 1999) was also performed to further analyze changes in community structure at a higher phylogenetic resolution. Samples for DNA were collected at the end of the experiments, after bacteria had reached maximum abundances, for one replicate of each temperature treatment by gently filtering at least 500 mL through 3 and 0.2 μm 47 mm diameter polycarbonate filters (Millipore) with a peristaltic pump. The 0.2 μm filters were frozen at -80°C until further processing. In some months, a decline in bacterial abundance was observed at the end of the experiments, likely associated to predation or cell lysis. Only DNA samples that had been collected before the drop in bacterial abundance were further analyzed by ARISA, in order to avoid the potential effect of predation in community structure, resulting in a smaller fraction of the experiments as compared to CARD-FISH. The ARISA method targets the differences in interegenetic ribosomal RNA regions that are highly heterogeneous but well conserved for different species (Ruan et al., 2006), therefore allowing for high resolution fingerprinting of microbial communities. We used the primers ITSF-Fam and ITSR-Reub designed by Cardinale et al. (2004) for the amplification of the 16S-23S rRNAintergenic transcribed spacers. DNA was extracted using the PowerWater DNA isolation kit (Mobio) and amplified by PCR with an initial denaturation at 94°C for 2 min, followed by 32 cycles of 94°C for 15 s, 55°C for 30 s, 72°C for 3 min, and a final extension at 72°C for 9 min. For PCR, 1 μL of sample (at 10 ng μL^-1^) was added to 39 μL of a master mix containing PCR buffer (4 μL), MgCl2 (2 μL at 50 mM), dNTP (1 μL at 10 mM), reverse and forward primers (1 μL at 10 μM of each), BSA (5.3 μL at 300 ng μL^-1^) and Phusion High Fidelity Taq polymerase (Biolabs; 0.6 μL at 5 U μL^-1^). For PCR product purification we used the QIAquick PCR Purification Kit (Qiagen). We performed capilar electrophoresis of the purified PCR samples with the size standard LIZ1200 (Applied Biosystems). Analysis of the electrophoresis peaks and fluorescence was done with GenMapper v4 (Applied Biosystems) and further binning of OTUs was performed using the R functions developed by Ramette (2009). Sizes of fragments ranged between 100 and 1000 bp and the relative fluorescence intensity cutoff value was set at 0.09 to exclude background noise. Window size for the bins was empirically established at 3 bp with a shifting window strategy of 0.1 bp. A total of 164 OTUs were identified in the 20 samples.

The structural composition distances between samples extracted using both CARD-FISH and ARISA techniques were analyzed using distance clusters (dendrograms) fed with Bray–Curtis dissimilarity matrices using the R package *vegan* (Oksanen et al., 2015) and *dendextend* (Galili, 2015).

## Results

**FIGURE 1:**
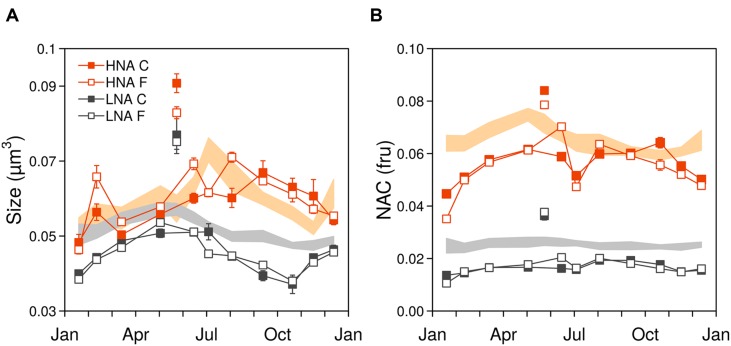
**(A)** Seasonal variation of bacterial cell size and **(B)** nucleic acid content (NAC). Data points represent the 2012 ambient values (mean ± SE), with closed and open symbols representing the community (C) and filtered (F) treatments, respectively. Shaded areas represent the 10 years time series mean ± SE values. May values were higher than usual, note the disconnection to the rest of 2012 values (fru, fluorescence relative units).

**FIGURE 2:**
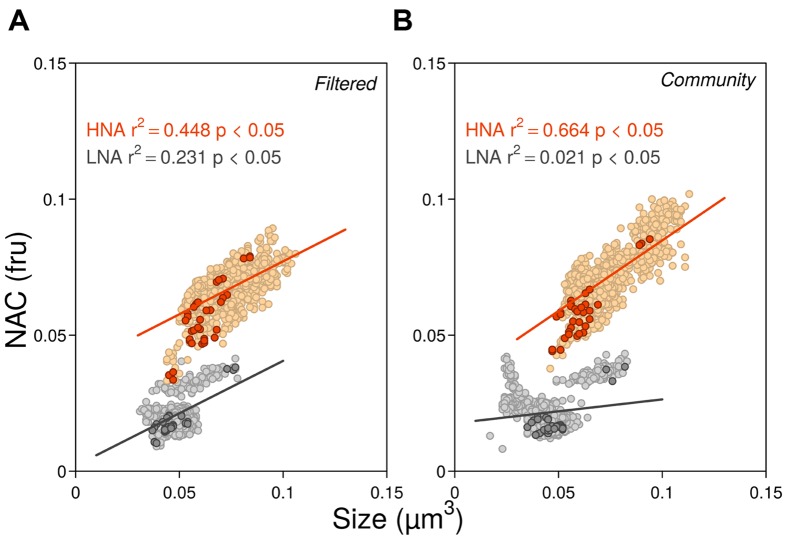
**Relationships between NAC and cell size for all experimental data in 2012 in the filtered (A) and community treatments (B).** Darker symbols represent initial ambient values while lighter symbols represent the rest of experimental data during the incubations. In both low nucleic acid (LNA) and high nucleic acid (HNA) groups, the largest and most fluorescent data correspond to the values of May. Ordinary least squares linear regressions included all data points (fru, fluorescence relative units).

The seasonal variations of ambient bacterial cell sizes and NAC are shown in **Figure 1**, together with the mean monthly values for the previous 10 years. May 2012 clearly departed from the seasonal pattern with very high values of both variables. Overall, cell sizes (**Figure 1A**) ranged from 0.035 to 0.091 μm^3^ and were consistently higher in HNA cells than in LNA cells, with values more similar around March–April, coincident with the pattern emerging from the decadal study. Filtration through 0.8 μm had an overall small effect upon the sizes of both groups (paired *t*-tests *p* > 0.05, *n* = 12) but did produce a shift toward larger sizes of HNA cells in three particular months (February, June, and August). In 2012 larger HNA cells were found in summer while larger LNA cells appeared in winter-spring. These patterns also matched the 10 years time-series seasonality (Morán et al., 2015), but the values diverged for HNA cells during the second part of the year with bigger sizes than the decadal mean while LNA cells were consistently smaller year-round. As with the decadal trend, over the year, HNA cell sizes covaried with temperature (*r* = 0.80; *p* < 0.05; *n* = 11). NAC ranged from 0.01 to 0.09 fluorescence relative units, with both LNA and HNA values generally lower than the corresponding 10-year monthly averages (**Figure 1B**). Overall, filtration had a negligible effect on NAC. The NAC of HNA cells was on average 70% higher than in LNA cells, and the respective coefficients of variation were the same (14%). With all experimental data pooled, bacterial size, and NAC were significantly correlated, although the relationships were stronger for HNA than for LNA cells (**Figure 2**). It should be noted that the data presented in **Figures 1** and **2** are the direct flow cytometry cell measurements, whilst the subsequent figures represent the slopes of NAC and cell size change with temperature.

**FIGURE 3:**
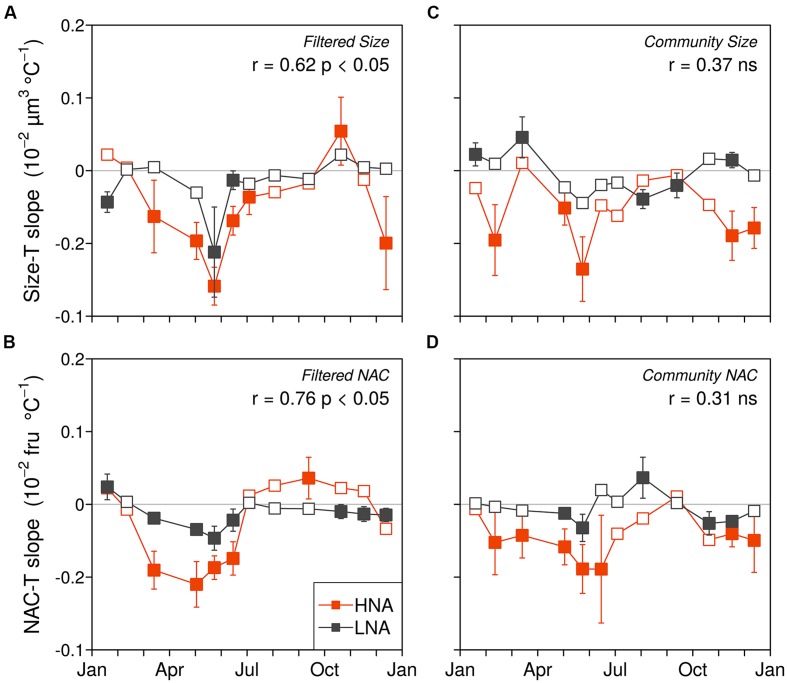
**Monthly variations in HNA and LNA cell size and NAC responses to temperature in the filtered (A,B) and community treatments (C,D).** Closed and open symbols represent non-significant and significantly different from zero OLS slopes with a 95% confidence interval. The Pearson correlation coefficients and significance between the HNA and LNA datasets for each variable and treatment are included in the panels (fru, fluorescence relative units).

**FIGURE 4:**
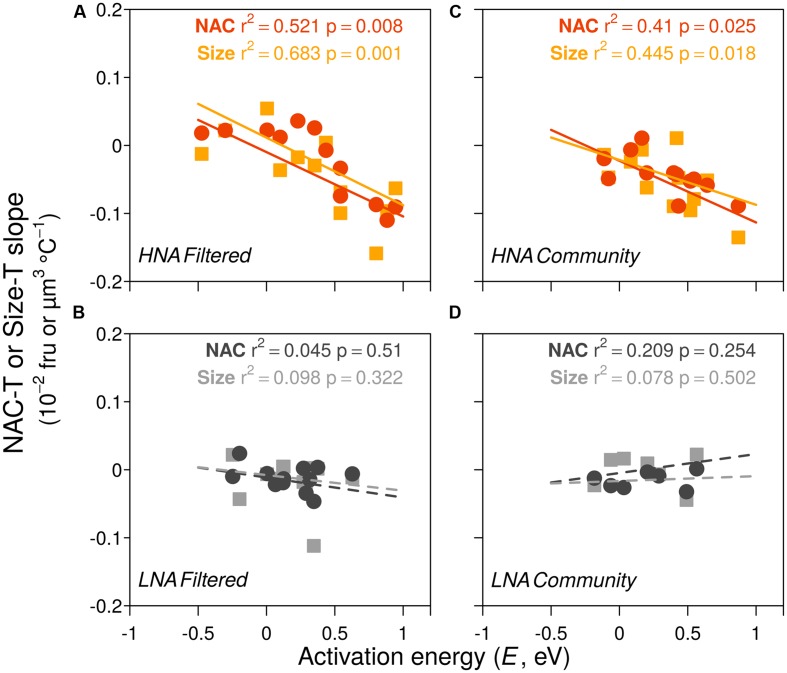
**Relationships between cell size-temperature slopes and NAC-temperature slopes versus activation energies (a measure of the temperature effect on growth rate) in the two groups of bacteria in filtered (A: HNA cells, B: LNA cells) and community treatments (C: HNA cells, D: LNA cells).** Dashed lines indicate non-significant slopes (fru, fluorescence relative units).

The cell size and NAC of the HNA and LNA bacterial groups generally decreased with temperature (i.e., more negative than positive linear regression slopes) in both the filtered (F) and community (C) incubations, although the overall effect was more marked for the HNA group, especially within the C treatment (**Tables 1** and **2**; **Figure 3**). The HNA bacteria changes in size and NAC with temperature were significantly correlated in both treatments (**Table 2**) while those of LNA cells were not. Regarding the covariation of HNA and LNA cells temperature responses, we observed strong correlations in the F treatment both for size (**Figure 3A**) and NAC (**Figure 3B**). The period of stronger decrease in size and NAC of bacterial communities with temperature consistently took place between February–March and June–July for both treatments. Spring to early summer was also the period of the year when we detected the strongest positive effect of experimental warming on bacterial growth rates (Huete-Stauffer et al., 2015). Indeed, the values of HNA bacteria cell size and NAC reduction per °C covaried with the corresponding temperature-dependences of the growth rates expressed as activation energies in both treatments (**Figure 4**). When warming did not result in increased growth rates (i.e., activation energies <0), the reduction in cell size and NAC was generally weak or even reversed (increased rather than decreased).

**Table 1:** Mean annual cell size and nucleic acid content (NAC) decrease per °C increase in temperature (fru, fluorescence relative units).

	Size (μm^3^ °C^-1^)	NAC (fru °C^-1^)
HNA F	-4.2 ± 1.7 × 10^-3^	-2.2 ± 1.5 × 10^-3^
LNA F	-1.6 ± 1.0 × 10^-3^	-1.2 ± 0.5 × 10^-3^
HNA C	-5.3 ± 1.2 × 10^-3^	-4.4 ± 0.8 × 10^-3^
LNA C	-0.5 ± 0.7 × 10^-3^	-0.4 ± 0.6 × 10^-3^

**Table 2:** Pearson correlation coefficients matrix between size reduction with temperature (Size-T slope in μm^3^ °C^-1^) and NAC reduction with temperature (NAC-T slope in fru °C^-1^) for both flow cytometry groups (HNA and LNA) and filtration treatments (C and F).

		Size-T slope				NAC-T slope			
		HNA F	HNA C	LNA F	LNA C	HNA F	HNA C	LNA F	LNA C
Size-T slope	HNA F	1							
	HNA C	-	1						
	LNA F	0.62^∗∗^	-	1					
	LNA C	-	-	-	1				
NAC-T slope	HNA F	0.77^∗∗^	-	-	-	1			
	HNA C	-	0.63^∗∗^	-	-	0.72^∗∗^	1		
	LNA F	0.79^∗∗^	-	-	-	0.76^∗∗^	-	1	
	LNA C	-	-	-	-	-	-	-	1

*Fru, fluorescence relative units. ^∗∗^ p < 0.01.*

**FIGURE 5:**
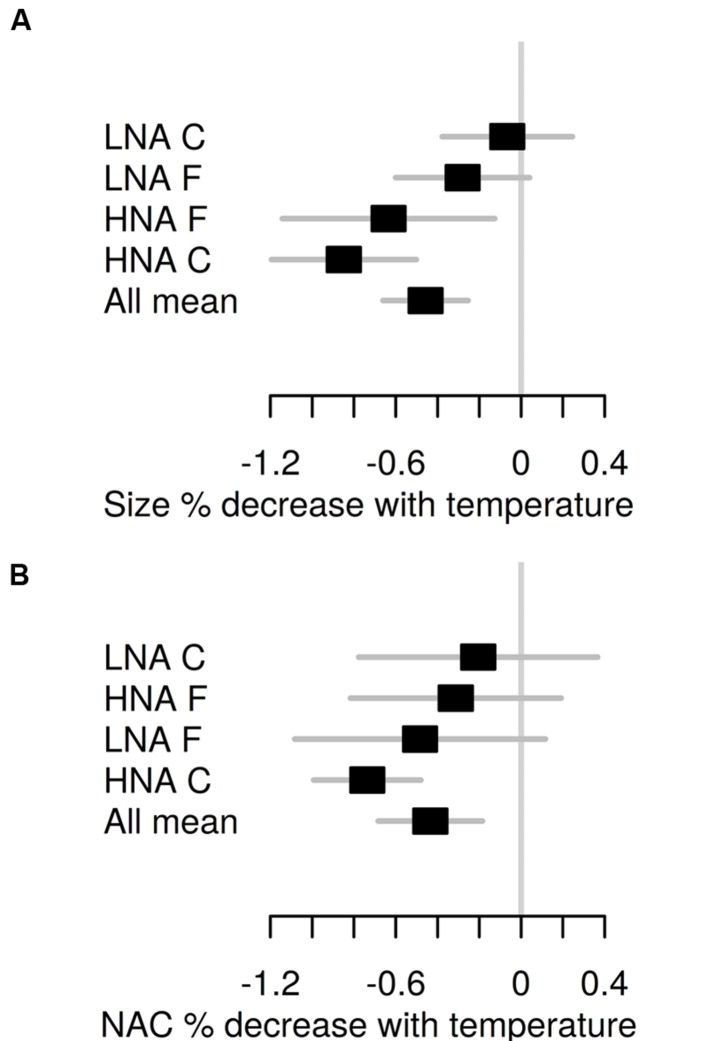
**Annual mean ± SE percent decrease in cell size (A) and NAC (B) per °C increase in temperature for each flow cytometric bacterial group and treatment ranked by increasing values.** “All mean” represents the average value for all groups and treatments.

The monthly percent reduction in cell size and NAC for a 1°C temperature increase (data not shown) closely followed the absolute values shown in **Figure 3**. The annual mean temperature-associated reductions for the two bacterioplankton groups at each treatment are shown in **Figure 5**. Mean reduction in bacterial size equaled the mean reduction in NAC at 0.45 ± 0.10%°C^-1^. HNA bacteria in the C treatment exhibited the largest reduction both in cell size and NAC, 92 and 72% higher, respectively, than the corresponding LNA bacteria values. However, in the F treatment the LNA cells percent NAC reduction was on average higher than that of their HNA counterparts due to the consistent increases rather than decreases observed in HNA cells for the second half of the year (**Figure 3B**). The equivalence of NAC reduction in base pairs units represented a mean loss of 26 kbp for HNA cells and 7 kbp for LNA cells for a 1°C increase.

**FIGURE 6:**
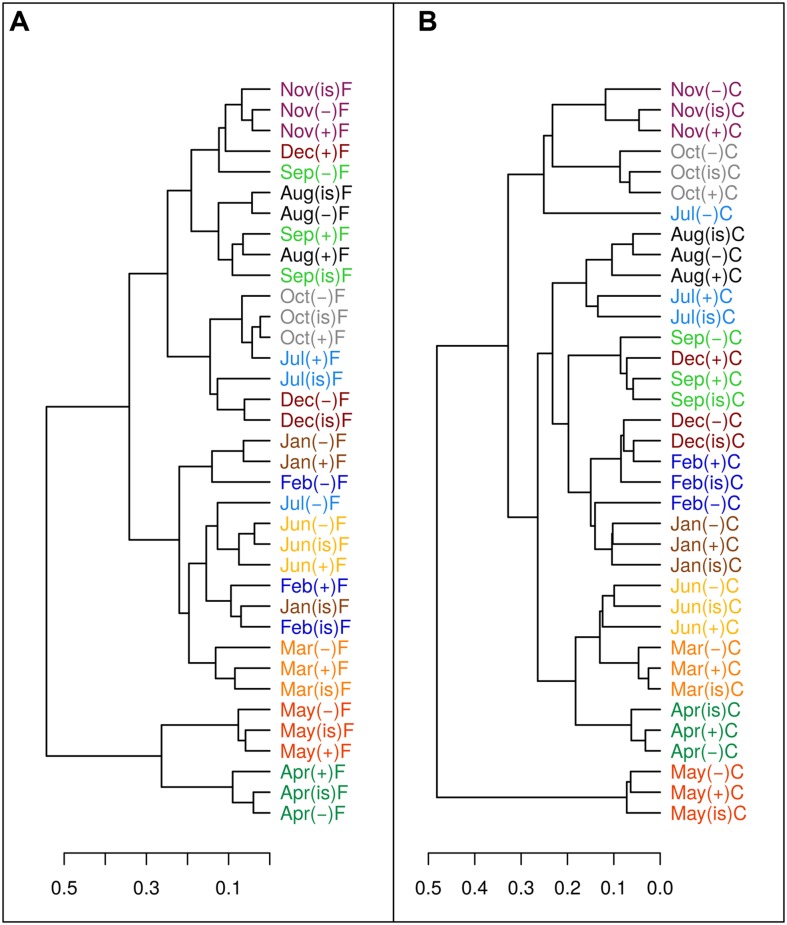
**Dendrogram of Bray–Curtis community structure distances using as proxy the relative percentages of abundances of four broad phylogenetic groups (SAR11, Rhodobacteraceae, Gamma-proteobacteria, and Bacteroidetes) determined by CARD-FISH, in the filtered (A) and community (B) treatments.** Months are color coded to facilitate interpretation. (“is” = *in situ*, “-” = -3°C, “+” = +3°C).

**FIGURE 7:**
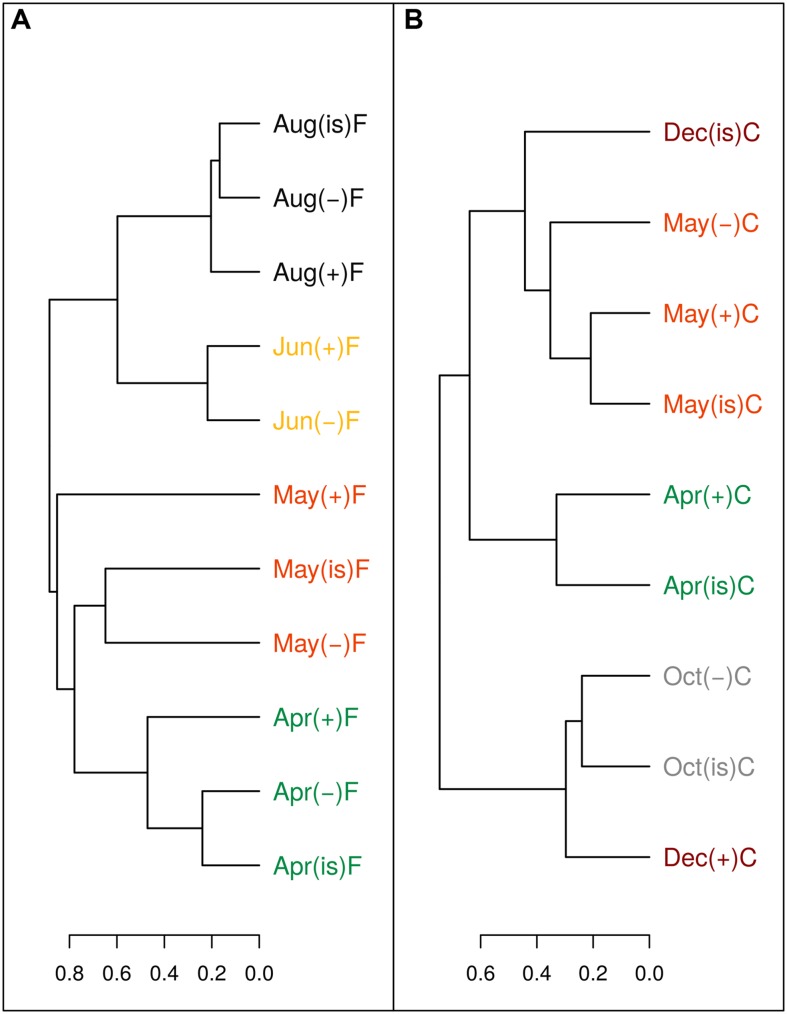
**Dendrogram of Bray–Curtis community structure distances of OTUs detected by ARISA for some of the experimental incubations in the filtered (A) and community (B) treatments.** Months are color coded to facilitate interpretation. (“is” = *in situ*, “-” = -3°C, “+” = +3°C).

The sum of the relative contributions of the four major taxa (SAR11, *Rhodobacteraceae*, Gammaproteobacteria, and Bacteroidetes identified by CARD-FISH, Pernthaler et al., 2002) for each sample, represented on average 70.4 ± 0.8% of the whole bacterial community identified by DAPI (Supplementary Figure S2). The contribution of each group changed during the experiments; for instance SAR11 usually decreased in importance while *Rhodobacteraceae* and Bacteroidetes generally grew (Arandia-Gorostidi *personal comm.*). However, samples from the same month and filtration treatment in the three temperature treatments generally clustered together (**Figure 6**), based on the similarity in community composition (i.e., relative percentages of the four major taxa, Supplementary Figure S2). Therefore, the effect of temperature on community structure at a broad phylogenetic level was limited. A higher resolution fingerprinting of the microbial communities, performed using the ARISA technique, overall corroborated the CARD-FISH results with a similar dendrogram output (for the 20 samples considered, **Figure 7**) of the binned distribution of the 164 detected OTUs (Supplementary Figure S3). With the exception of three samples (December in the C treatment and the +3°C sample of May in the F treatment), all temperature treatments of each experiment tended to cluster together, suggesting limited changes in community composition at the level of resolution offered by ARISA.

## Discussion

To study the response to temperature of the cell size and NAC of heterotrophic bacterioplankton we relied on the respective flow cytometric signals of right angle light scatter or side scatter (SSC) and green fluorescence after Sybr-Green I staining. SSC was used as the cell size proxy instead of forward angle light scatter because of its better sensitivity when analyzing slight differences in the volume of the smallest aquatic microbes (Gasol and Morán, 1999; Felip et al., 2007; Wang et al., 2009; Müller and Nebe-von-Caron, 2010). The Sybr-Green I fluorochrome was chosen to quantify NAC because of its affinity for a variety of nucleic acids including dsDNA, ssDNA and even ssRNA (Brussaard et al., 2000). It should be noted that the side scatter and green fluorescence variables are measured independently by different detectors in a flow cytometer (Rothe, 2009). However, we found a positive covariation between the two of them (*r* = 0.82, *p* < 0.001, *n* = 4353, all data pooled) indicating that cell size and NAC are strongly associated in coastal marine bacteria, as previously demonstrated in thermophilic bacteria (Sabath et al., 2013) and also multicellular organisms (Gregory, 2001; DeLong et al., 2010; Hessen et al., 2013). Although widespread, the origin of the cell size and genomic material correlation, e.g., co-evolution or coincidence, is not clear (Gregory, 2001), but as shown in this study, temperature may have a role in controlling both the cell size and the NAC in marine bacterial assemblages. Yet, it should be noted that the experimental reductions in bacterial cell size and NAC showed here occurred in short incubations (days), and therefore the mechanisms involved are likely different from evolutionary forces on both cell and genomes sizes reported for some marine bacteria (Giovannoni et al., 2005), as discussed below.

The annual seasonality of cell size in the upper mixed layer at the study site has been characterized in a 10 years study ending in March 2012 (Morán et al., 2015). Data for NAC were also available for the same period and are first shown here (**Figure 1**). Overall, the initial values of cell size and NAC of surface bacteria collected for the monthly experiments were in accordance with the seasonality that emerged from the decadal time-series (**Figure 1**). In 2012, the sizes of the HNA and LNA cells followed the patterns predicted in the latter study, although the size of LNA cells was lower (paired *t*-test *p* < 0.01, *n* = 11, excluding May). Two key periods were differentiated in the seasonality of these parameters: winter-early spring (January to April) when LNA and HNA sizes were more similar (0.047 ± 0.001 vs. 0.051 ± 0.001 μm^3^, respectively) and summer-early autumn (June to October), which was characterized by large HNA cells and small LNA cells (with a peak in August/September, when HNA cells were 56% larger than LNA cells). Regarding the seasonality of the NAC of the bacterial cells, our initial values were usually below the mean of the previous years (**Figure 1B**, see also Figure 4D in Huete-Stauffer and Morán, 2012). However, the reduction of the cell size and NAC values in comparison with mean values of the previous decade, would be consistent with the significant temporal decrease in the cell size found for both HNA and LNA cytometric populations at the site of study (Morán et al., 2015).

The large differences observed in mean NAC between the HNA and LNA clusters support the view that the two widespread cytometrical groups are consistently made up of different taxa (Schattenhofer et al., 2011). HNA cells are likely more heterogeneous and composed by seasonally succeeding bacterial phylotypes with larger genome and cell sizes. On the contrary, LNA cells are likely dominated by small and genome-streamlined bacteria, such as the SAR11 clade (Grote et al., 2012), with seasonally less variable cell and NAC values. Indeed, at our site, a significant correlation between LNA cells and SAR11 abundance has been reported (Morán et al., 2015). As for HNA bacteria, we found that HNA cell sizes covaried with temperature (**Figure 1A**), likely due to the seasonal phylogenetic succession of HNA populations in response to environmental conditions.

In the experiments, in order to isolate the temperature effect on the cell size and NAC of bacterial assemblages from other potentially disturbing factors, we analyzed two treatments: one preserving the whole microbial community (C) and one pre-filtered by 0.8 μm (F), which excluded virtually all larger planktonic members (including phytoplankton and protistan grazers). HNA and LNA cells in the F treatments followed very similar seasonal patterns in their responses of cell size to temperature (**Figures 3A,B**) but this relationship was sloppy in the C treatment, where community interactions (e.g., size-selective grazing and/or phytoplankton primary production) could have obscured the direct effect of temperature. Although cell reduction affected both LNA and HNA bacterial communities, these changes were consistently smaller in LNA cells relative to HNA cells. This supports the idea that LNA cells, more homogeneous in phylogenetic composition, and likely dominated by members of the SAR11 clade (Morán et al., 2015), have a more limited ability to decrease in size in response to warming, given the already extremely small volumes (Rappé et al., 2002). However, it should be noted that a significant decrease in LNA cell size (equivalent to 1.74% per °C) was detected after 10 years of sustained observations in ambient waters (Morán et al., 2015). Interestingly, this decrease in LNA cell size was smaller than that of their HNA counterparts at the decadal scale (4.10% °C^-1^). This coherent difference in the behavior of the two groups strongly suggests that future warming-associated reductions in size will preferentially target HNA bacteria.

The period of the year between late winter to early summer (March to June–July), characterized by meso- to eutrophic conditions, is ecologically relevant at our study site because it comprises most of the spring phytoplankton blooms and the subsequent progress of stratification, including maximum annual growth rates of bacterioplankton assemblages (Franco-Vidal and Morán, 2011; Huete-Stauffer et al., 2015). Additionally, it represents a consistent period of temperature increase observed over the previous 10 years (ca. 1.5°C warming: Morán et al., 2015), and thus, it seems to be the most sensitive to warming. Remarkably, during this period, consistent decreases in cell size were observed in the experiments. In these same months we had observed the greatest positive temperature dependences of HNA cells growth rates (i.e., activation energies close to 0.65 eV: Huete-Stauffer et al., 2015), with seasonal patterns very similar to those of cell size decrease. Indeed, the temperature-associated reductions in size were strongly correlated to the increases of HNA cells growth rates with warming, more marked in the filtered (**Figure 4A**) than in the non-filtered samples (**Figure 4C**). Particularly in the F treatment, changes in activation energy explained 68% of the variance in cell shrinkage of HNA cells in response to temperature rises, suggesting a link between both processes. Indeed, shrinkages in organism sizes have been related to increased growth rates and surface to volume ratios, both in turn positively related to higher temperatures (Angilletta et al., 2004; Savage et al., 2004; Kingsolver and Huey, 2008). In addition, since respiration increases exponentially with temperature regardless of the possible effects of nutrient limitation on production (Yvon-Durocher et al., 2012), bacterial cells allocation of proportionally more carbon into respiration than into cell enlargement (Rivkin and Legendre, 2001; Wohlers et al., 2009) would enhance cell size shrinkage. If, as historical data suggest (Morán et al., 2015), ocean warming will continue with the same pattern of marked increases in temperature in spring, together with higher mean annual abundances of marine bacterioplankton as a result of growth rate increases (Huete-Stauffer et al., 2015), we will likely record smaller sizes of marine bacteria in the future.

Remarkably, the changes in NAC of marine bacterioplankton in response to environmental factors (including seasonal patterns) have not received much attention so far. Interestingly, we found a consistent decrease of NAC of bacterial communities in the experiments in response to temperature, following the same patterns described for cell size (**Figures 3B,D** and **4**). The mean annual rate of temperature-associated decrease in both cell size and NAC was the same when we pooled bacterial groups and treatments (**Figure 5**), furthermore supporting the linkage between cell size and NAC in bacterial communities. The observed decrease of 0.4% °C^-1^ in our experiments is nevertheless lower than the 2.5% °C^-1^ decrease in body size proposed by Atkinson et al. (2003) for protists, and especially much lower than the 10% reduction in bacterial cell size observed by Morán et al. (2015) in the mentioned environmental decadal study (but it should be noted that this value was coincident with Atkinson et al. (2003) value of 2.5% °C^-1^ when it was directly related to annual temperature changes).

At the evolutionary scale, genome reduction in thermophilic bacteria has been related to temperature through increased growth rates and reduction of intergenetic regions (Sabath et al., 2013). However, reductions in genome size most likely escaped the short duration of our experiments. The most parsimonious explanation for the consistent decrease in cell size and NAC with temperature would involve changes in community structure during the incubations at different temperatures such that increasing temperatures may have promoted the growth of bacterial phylotypes with smaller genomes and cell sizes, in accordance with the idea of smaller species selection at higher temperatures (Daufresne et al., 2009; Kerrigan et al., 2015). Ancillary phylogenetic data based on CARD-FISH and ARISA show mixed results in this regard. While substantial changes occurred in a few particular treatments and experiments, in general there were no major taxonomic shifts at the different temperature treatments at the broad level of resolution offered by CARD-FISH (**Figure 6**; Supplementary Figure S2), particularly during the period from March to June when the changes in size and NAC were more pronounced. These results were confirmed at a finer taxonomic resolution in some experiments using ARISA (**Figure 7**; Supplementary Figure S3). For example, filtered samples from April to June clustered together (due to very high similarity in the OTU profiles, Supplementary Figure S3) when a substantial decrease in NAC was detected (**Figure 3**). However, changes in community structure were observed in filtered incubations in May and in unfiltered samples in December (as also shown by CARD-FISH). In summary, our results show that changes in NAC and cell size were in some cases accompanied by changes in the phylogenetic composition of bacterial communities, but substantial changes in NAC and size were also detected in incubations where bacterial community structure remained rather similar. However, there are limitations to the techniques used for detecting changes in bacterial community composition. On the one hand, CARD-FISH targeted only broad bacterial groups, which can include members with very variable cell and genome sizes; on the other hand, ARISA detects preferentially the most abundant taxa (~ top 30%, Gobet et al., 2014), likely overlooking shifts in less abundant groups or in groups where the 16S and 23S are not found in a single operon (Kovacs et al., 2010). Furthermore, variability in the genome size of selected species with identical ARISA profiles may occur (Kovacs et al., 2010). Thus, we cannot fully exclude the possibility that shifts in bacterial populations with different genome size explained the decrease in NAC in our experiments, even in those cases where bacterial community structure remained similar.

On top of the explanation by shifts toward phylotypes with smaller genomes, some additional aspects can be considered to help explain the reduction in NAC content in the experiments, for instance, loss of RNA or extra chromosomal nucleic acids such as plasmids. Ribosomal RNA is one of the major components in bacterial cells (Neidhardt and Umbarger, 1996) and the rate of rRNA synthesis changes rapidly in response to changing environmental conditions (Molin and Givskov, 1999). As the fluorochrome used (Sybr Green I) also stains RNA (although with less affinity than DNA, Brussaard et al., 2000), changes in NAC content may have been partly caused by changes in RNA content. Even if fast growing cells typically contain more rRNA (Elser et al., 2003), increases in rRNA content at lower temperatures have been observed in phytoplankton cultures (Toseland et al., 2013), apparently associated to the need to produce more ribosomes to compensate for the reduced translation efficiency. It would be interesting to test whether this mechanisms is also relevant for marine bacteria. Alternatively, the loss of plasmids at higher temperature could also produce a decrease in NAC, as higher growth rates imply shorter intergeneration times where replication of plasmids becomes a secondary task (Mosrati et al., 1993). As plasmids often carry auxiliary genes that may improve cellular fitness or allow for horizontal gene transfer (Ma et al., 2012) their systematic loss with warming may have consequences for the phenotypes of the bearing organisms. Interestingly, roughly converting NAC reduction into base pairs units, the amount of DNA lost per °C would reasonably agree with the size range of plasmids (7–26 kbp °C^-1^) in marine bacteria (Sobecky et al., 1997; Leitet et al., 2006). However, none of the above mentioned mechanisms of NAC reduction could be proved with our data, and they represent hypotheses that could be tested in further studies.

As in every experimental study using batch incubations of seawater, the manipulation and bottle confinement may have induced biases in community composition (Fuchs et al., 2000) so that our results may not have reflected accurately the natural responses. However, experimental manipulation is difficult to avoid when the effect of a particular variable such as temperature needs to be isolated from other factors. Normally the so-called “bottle effect” includes growth of certain groups with the ability to attach to surfaces, changing the composition of the natural assemblage. Typical groups that prefer surface attachment and are stimulated in confinement are *Rhodobacteraceae* and Bacteroidetes (Dang and Lovell, 2016), which also have larger sizes and genomes than typical free-living bacteria such as SAR11. It could be argued that our sampling design selected for smaller free living bacteria (overlooking larger attached cells) but nonetheless, the same bias would equally apply to all temperature treatments, therefore affecting little the observed experimental changes in bacterial size and NAC.

In any case, the pervasive finding of bacterial cell reduction both at short (this study) and long (Morán et al., 2015) time scales in seeming response to rising temperatures, based on experimental and observational data, respectively, is hard to neglect. The demonstrated association between cell size and NAC and their changes with warming in coastal bacteria, constitute highly relevant results in terms of elucidating the underlying processes and the consequences that a widespread size reduction and nucleic acid loss in marine bacteria will bring for the ecology and biogeochemistry of the future ocean.

## Author Contributions

TH-S and NA-G contributed equally to the experimental setup and design, field and laboratory work, as well as to data acquisition. NA-G processed all CARD-FISH data. TH-S additionally processed and interpreted the flow cytometry data and wrote the manuscript. LA-S guided all molecular techniques and as XAGM, contributed in designing and supervising all experimental work as well as in data interpretation and manuscript preparation and revision.

## Conflict of Interest Statement

The authors declare that the research was conducted in the absence of any commercial or financial relationships that could be construed as a potential conflict of interest.
