# Supplementary material for: Experimental Warming Decreases the Average Size and Nucleic Acid Content of Marine Bacterial Communities
Source: Front Microbiol. 2016 May 23;7:730. doi: 10.3389/fmicb.2016.00730 (PMC4876119; doi:10.3389/fmicb.2016.00730)
Supplement: Supplementary file 1 [file Image_1.PDF]

Supplementary Figure

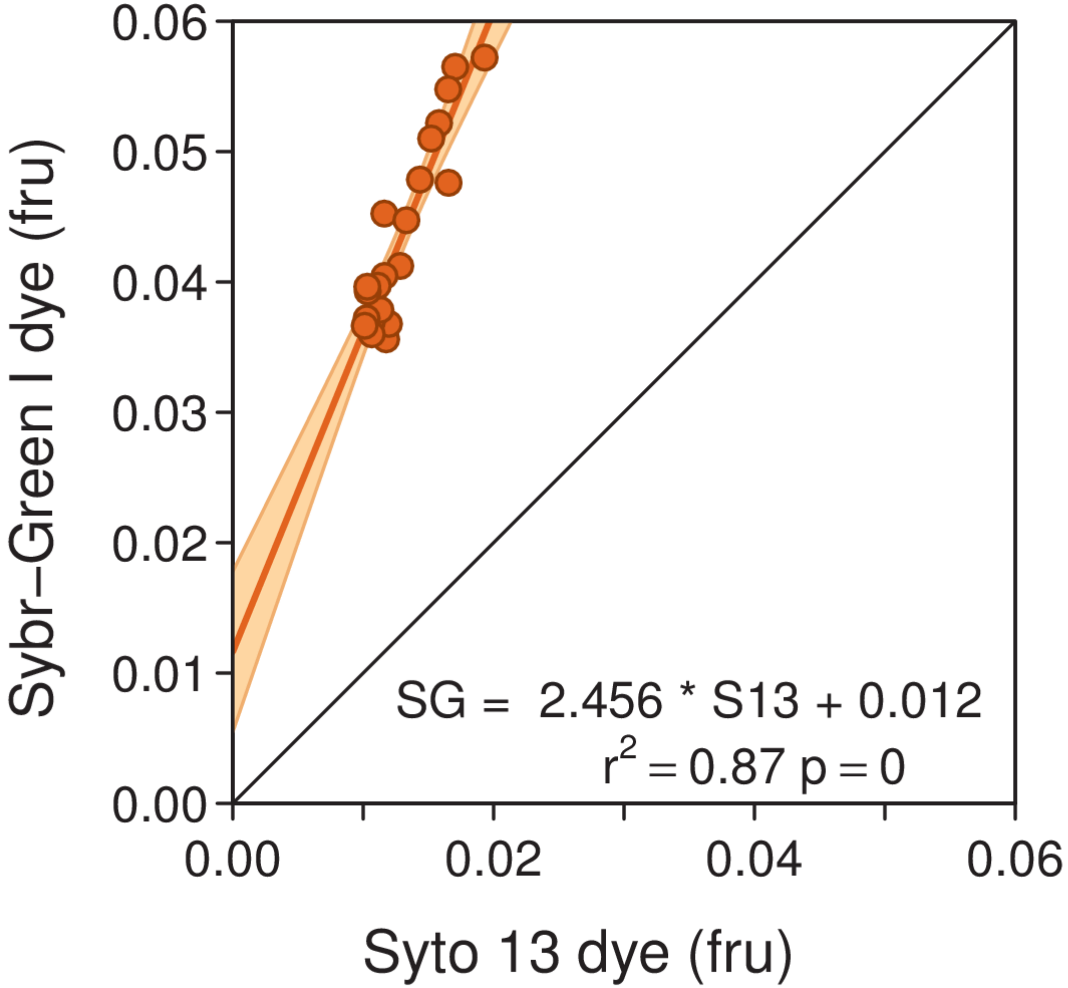

Fig. S1. Empirical calibration used to transform nucleic acid fluorescence in samples stained with Syto 13 to Sybr-Green I values in order to compare the 2002-2012 decadal mean and the 2012 experimental values shown in Fig. 1.
