# Supplementary material for: Experimental Warming Decreases the Average Size and Nucleic Acid Content of Marine Bacterial Communities
Source: Front Microbiol. 2016 May 23;7:730. doi: 10.3389/fmicb.2016.00730 (PMC4876119; doi:10.3389/fmicb.2016.00730)
Supplement: Supplementary file 2 [file Image_2.PDF]

Supplementary Figure 2

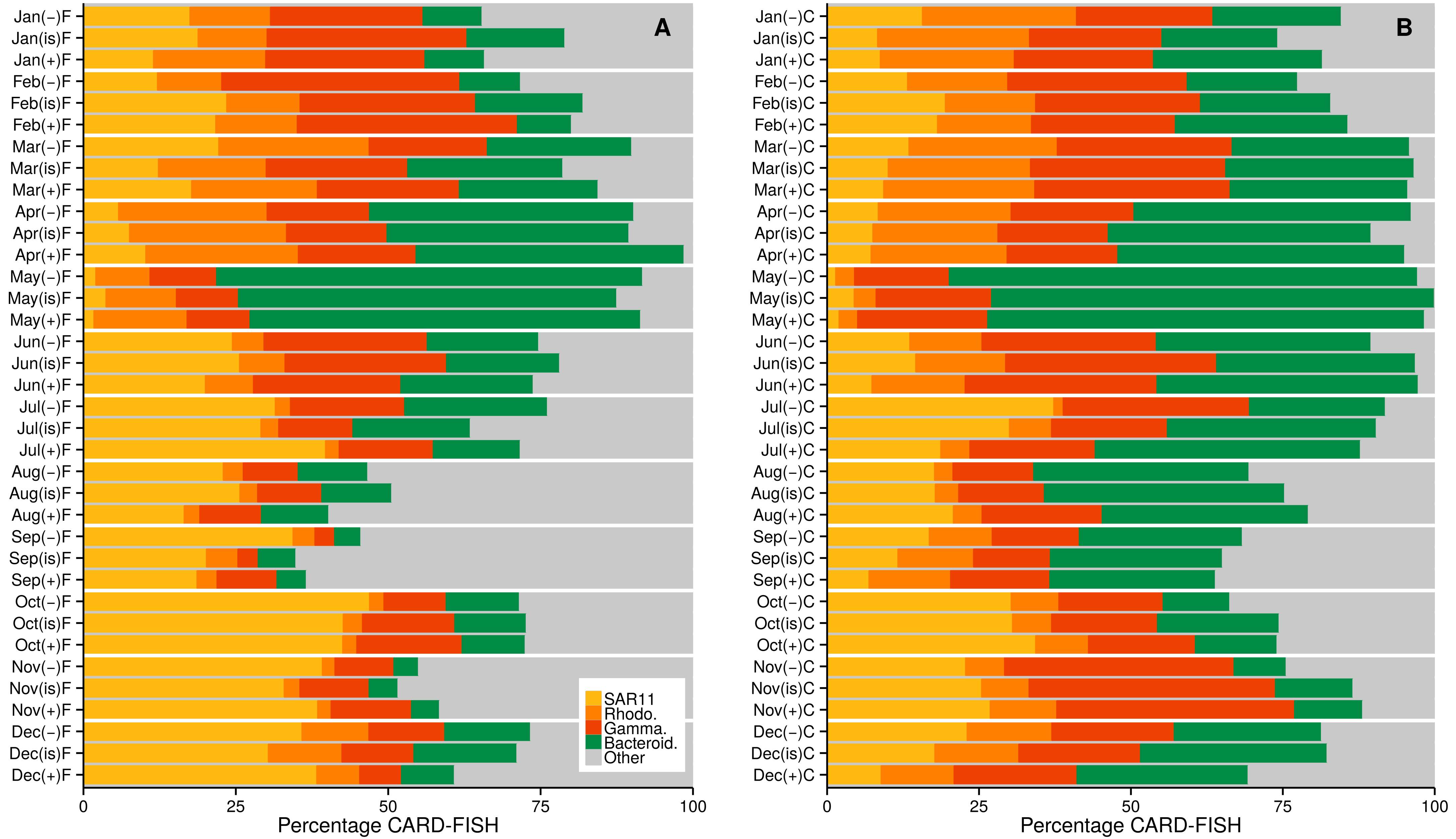

Fig. S2. CARD-FISH percentage abundances of 4 broad phylogenetic categories at the final growth stage of all the experimental warming incubations in the filtered (A) and community (B) treatments.
