# Supplementary material for: Experimental Warming Decreases the Average Size and Nucleic Acid Content of Marine Bacterial Communities
Source: Front Microbiol. 2016 May 23;7:730. doi: 10.3389/fmicb.2016.00730 (PMC4876119; doi:10.3389/fmicb.2016.00730)
Supplement: Supplementary file 3 [file Image_3.PDF]

Supplementary Figure 3

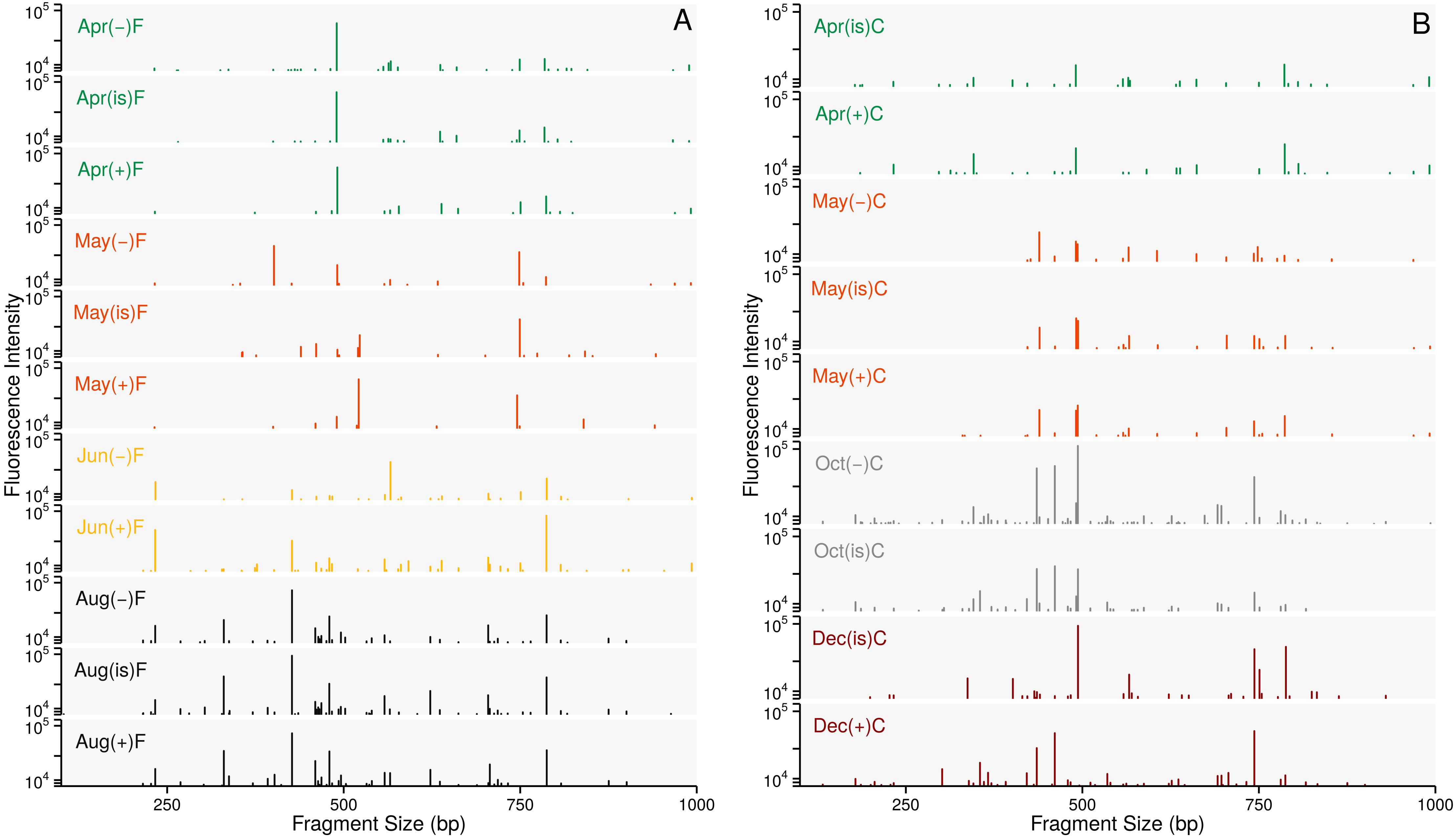

Fig. S3. Distribution of OTUs detected by ARISA for a reduced number of samples in the filtered (A) and community (B) treatments.
